# Bolton Infirmary Inquiry

**Published:** 1910-07-16

**Authors:** 


### Hospitals of the United Kingdom.

### BOLTON INFIRMARY INQUIRY.

### FULL TEXT OF DR. MACKINTOSH'S REPORT.

The *Bolton Journal* of July 9 contains the following :—

The monthly meeting of the Board of Management of the Bolton Infirmary was held yesterday afternoon, under the presidency of Mr. John Harwood, J.P., when the report was read of Dr. Mackintosh, M.B., M.V.O., F.R.S.E., medical superintendent of the Western Infirmary, Glasgow, on his visit to the institution. It will be recalled that Sir Henry Burdett, in The Hospital [p. 574, February 12, 1910], passed a criticism on the institution. In his article Sir Henry said the Infirmary suffered from the absence of adequate ventilation and strict administrative efficiency; the wards contained too many beds and lacked a through current and interchange of air. Reference was made to the large number of operations conducted in a single day, and it was stated that "the children's wards were some of the saddest we have ever inspected." The critique concluded : "Our impression was that the whole of the Bolton Infirmary would benefit by the introduction of a little outside influence and life. The whole establishment needs to be aroused and stirred up, and we direct the attention of the Committee to this criticism with full confidence that they will consider it on its merit." After considering the article, the Committee asked the British Medical Association to select some gentleman eminent in the profession to visit and report on the institution. Dr. Mackintosh was chosen by the Association to perform this duty. After the report had been read the Committee decided that it be published. The following is the complete text of the report, which is dated Western Infirmary, Glasgow, June 21, 1910 :—

In response to a request from you to visit the hospital and report fully and completely on its condition and administration, and accompany my report with any practical recommendations likely to increase the efficiency of the hospital, I visited the institution on June 3 at 4 p.m., being met on my arrival by representatives from your Committee and honorary medical staff. I thereafter proceeded to go over the building, accompanied by the senior house surgeon through the wards and by the matron through the domestic department and nurses' quarters. The secretary's department was left over till the forenoon of Saturday, June 4.

#### The Extensions.

The Infirmary was opened on October 23, 1883, twenty-seven years ago, and cannot therefore be regarded as an old building. It was at once apparent to me that in preparing the plans of the original buildings, the question of any possible future extension of the Infirmary being required had not received consideration. When it was found necessary to provide additions in order to meet increased demands on the institution, it seems to have been considered impossible to provide the increased accommodation for patients and staff without interfering with the light and air required for the separate buildings. The additions have been put up possibly in the most economic way, but they cannot be regarded as the best examples of modern hospital construction; in one case particularly they prevent the complete through-ventilation of a ward.

#### What the Doctor Found.

When I visited the hospital there were 137 patients in residence. The general wards are 60 ft. in length, 28 ft. in breadth, and 16 ft. high. Each ward has constantly eighteen occupied beds, although they were designed to accommodate fourteen. The number of accidents and urgent cases seeking admission is out of proportion to the present available accommodation, and unless the public come forward and subscribe a sum sufficient to justify the managers in making the necessary additions, it is difficult to see how overcrowding can be avoided. The question of increasing the accommodation for patients, however, involves other matters of importance which must be considered before there is any further extension of the wards. The beds in the wards are too close together, and, in consequence, there is not sufficient room for the necessary bedside furniture. The ward floors are completely worn out, and ought to be renewed at once. They were originally laid in white pine, and in one or two wards linoleum has been put over the worn-out floor, which covers up the defects, but in every case these floors should be taken up and laid with some hard wood, such as maple, teak, or pitch pine. The lockers, which serve as chairs, bedside tables, and cupboards for patients' clothes, have seen their day, and ought to be replaced by more modern fittings. The cabinets in the middle of the ward floors are much too heavy to be moved for cleaning purposes, and when the floors are renewed, these cabinets should either be made fixtures or removed entirely, and cabinets on wheels substituted. The service kitchens adjoining the wards are in some cases larger than usual, but in none of them are there cupboards for milk, etc., with ventilation to the open air. In one of the ward kitchens there is a cupboard containing splints. This should be removed from the ward kitchen, and a cupboard or racks fitted up for holding brushes, instead of having them lying on the floor, seeing it is not possible at present to provide a scullery or housemaid's closet adjoining the kitchen for this purpose in every case. There are wooden partitions at the ends of the wards which might be taken away provided the doors into the sanitary towers are put on proper spring hinges. The lavatory towers have wooden floors. These should be taken up and replaced by tiles or terrazzo. Wood is quite unsuitable for the floor of such an annexe. There is a sink in the floor for washing utensils, which is too low for practical purposes, and should be removed. If a swan-neck tap working on a swivel were put on the cold water supply pipe to the bed-pan washer, it could meantime take the place of the sink which is on the floor, and prove sufficient until such time as these fittings require renewal. There is no aired cupboard in this tower for keeping specimens until the doctor's visit. The window breast ought to be cut out and a proper cupboard inserted with iron doors to the inside, and a ventilating grating to the outside, and, in addition, racks should be supplied for holding the utensils instead of having them piled one over the other on the window-sill. Up-to-date fireclay baths have been put into some of the bathrooms, but I would suggest that when other baths require to be renewed an enamelled iron bath would serve the purpose, and be much less costly.

#### Cot Ward and Ventilation.

There is a ward called the "Cot Ward," which has no sanitary annexes adjoining. The nurse must pass through one of the other wards to get to bathrooms or lavatories. This ward, which is now used for children, was evidently originally designed as a day-room or recreation room for the two wards adjoining. If it is proposed to continue using this room for its present purpose, some provision should be made for a sanitary block being attached. On the front of the building at both ends there are small wards used for eye cases. There is not sufficient bathroom accommodation adjoining these wards, and some of the sanitary fittings require renewal. Every available corner seems to be made use of, and, in consequence, rooms which were intended in the original design for other purposes have been used for the accommodation of patients. The ward adjoining the main entrance, which is at present used as a women's ward, has been rendered less efficient by the new addition which has been placed alongside it. This ward was originally designed to have through ventilation, but the addition which has been added prevents the free current of air, and the new ward has not a sufficient supply of direct fresh air. I would suggest that air inlets, guarded by wire gauze, be cut in the wall above the floor level, opposite the radiators, for the admission of fresh air. I would further recommend that the windows, which are at present sealed between the one ward and the other, should be taken out, so that, as far as possible, cross ventilation may be secured. On the north portion of the site there is an isolation house. I understand it is seldom used, but if it came to be used frequently, it would be necessary to have a bathroom fitted upon the level of the wards. Quite recently telephones have been fitted up, giving intercommunication between the wards and various departments.

### PATIENTS WELL CARED FOR.

### Suggested Increase of Medical Staff.

I am satisfied that the patients are well cared for; the wards when I visited were tidy, and the beds scrupulously clean. The work of the house surgeons should be rearranged. At present it is very exacting, and I consider that additional assistance should be provided. I would recommend that one or two anæsthetists should be appointed. These do not require to be resident, but preferably practitioners in the town. A bacteriologist should also be appointed to do clinical research work. There is a small laboratory leading off the main corridor, which, in the meantime, would be quite suitable for the purpose. The addition of one or two assistant surgeons might also be considered. There are men with the necessary qualifications and experience in Bolton who might be willing to apply for such appointments. They would not only relieve the house-surgeons and allow them to attend to emergency and urgent work, but would, in my opinion, add materially to the efficiency of the establishment.

### The Operating Theatre.

The out-patient department is new, and seems to meet the requirements. The addition of a small operating room and recovery room to the accident and admission department would be a distinct advantage. The operating theatre is a comparatively new building, but could be considerably altered with advantage. It is really too large for the purpose, and the steriliser for dressings should be placed in an adjacent room, and not in the theatre. The rooms adjoining the theatre could be re-arranged, and there is abundant space for a sterilising room. The present arrangement of having some of the dressings prepared away from the theatre is open to criticism. All dressings should be prepared alongside the sterilising room, and afterwards distributed to the various wards and departments. The windows in the operating theatre should be made to open. Several of them are fixtures, and the coil placed out on the floor ought to be removed. At present it is leaking, and takes up far too much room. I understand that the theatre sister, or nurse, takes duty in the out-patient department. It may not be possible to alter this until the nursing staff is increased, but it should be kept in view.

### X-ray Room.

What was originally the operating theatre is now used as an *x*-ray room. The room where the coil is placed is rather small, but if the partitions were removed and placed further out it would be an improvement.

### Quarters for the Nurses.

The nurses' sitting-room leads off the main corridor of the hospital. It is not an attractive room, and is much too public. Their dining-room is a dull room, and lit only on one side by windows at the back of the buildings to the north. The pantry adjoining the dining-room is much too small, and should be converted into a larder for milk. I would suggest that one of the rooms in the main building facing the south, say the library, should meantime be used as a nurses' dining-room. A small service lift could be fixed up in the corridor on the wall opposite their present dining-room to convey the food. The present dining-room and doctors' pantry which adjoins it should be divided, and made into two good pantries, one for nurses and the other for doctors, as the present pantry and larder for the doctors are small. If the library is used for this purpose, the house surgeons can be transferred to similar rooms on the other side of the main staircase, but the details of this can be worked out later and shown on a plan. The semicircular corridor leading from the main corridor to the wards on the north portion of the site is much worn, and should be relaid or covered with red cement. One has only to see a patient being conveyed in an ambulance barrow over it to appreciate the urgent necessity of something being done. A series of nurses' rooms are constructed over this corridor. The entrance to these rooms is from a passage three feet wide. The rooms themselves are comfortable, but there are not enough of them, and before the managers consider the provision of additional accommodation for patients they must set their minds to the problem of providing additional and more up-to-date quarters for the nurses. At present four night nurses require to sleep in one room; some of the others are in double rooms. Each nurse should have a bedroom to herself, and suitable sitting-rooms should be provided.

### Nurses' Home Wanted.

This I realise will necessitate the building of a Nurses' Home; but if the general public and the wealthy classes of Bolton and neighbourhood fully appreciate the value of the work performed by the nurses, they will see that unless more adequate accommodation is provided for them, the Infirmary will not be able to command the services of the best class of nurses and probationers, as these ladies will naturally try to get their training where more up-to-date accommodation exists. The nurses' training in Bolton Infirmary is of a high standard, and the experience gained in the wards is excellent. The number of nurses at present on the staff might be increased with advantage, for it must always be kept in view that this is really a surgical hospital, the majority of the cases being acute. I would therefore urge on the Committee of Management the necessity of at once facing the question of securing funds to build a Nurses' Home. This need not be an elaborate or expensive building, but it is now fully appreciated by hospital authorities that if they are to continue to attract the right class of women to the nursing profession, comfortable quarters must be provided for them without the hospital proper. Some of the furnishings and fittings in the nurses' rooms require repair, and several of the floor rugs should be renewed. These may appear very small matters to take special note of, and as isolated examples I admit they are; but if allowed to accumulate, they strike at the root of efficiency. All minor repairs and renewals should be attended to without delay when the head of the department has pointed them out. Of course, all repairs and alterations involving considerable expenditure, and which are not urgent, should be brought before the House Committee at their first meeting, but I will suggest how this might be dealt with later. If a new Nurses' Home were provided, better accommodation could be found for the maids.

### In the Laundry.

The laundry, which is reached from the semicircular corridor leading to the wards on the north portion of the site, has sufficient machinery to carry on the work, but when I visited there was a very strong smell of gas emanating from the gas-irons. This is due to the fact that sufficient air is not being driven in to supply the burners. On looking into the matter, I found that the fan for supplying air to these gas-irons was driven from the laundry engine, which is so irregular in speed owing to the machinery being taken on and off, that the supply of air is not regular, nor is it sufficient for the purpose. An electric motor should be installed, with a suitable fan. This would at once do away with the smell of gas, and could be carried out at comparatively little cost. The laundry staff reside on the premises; four of them sleep in one room. It would be an advantage to have these workers accommodated in cubicles or bedrooms by themselves. The wages paid to the laundry workers compare favourably with similar workers in other institutions, and, in addition, I found that, as in all up-to-date institutions, they are supplied with uniform, which gives them a tidy appearance. Adjoining the boiler-house there is a room used for purposes of disinfection by sulphur. This room should be fitted up with steam pipes. Of course, all infected clothing due to fever or any such cause is efficiently dealt with by the public authorities.

### Kitchen and the Meat Supply.

The general kitchen is centrally placed, and seems to be considered sufficient for present requirements, although there is no hot plate or steam ovens. There is a lack of suitable larder accommodation; the stores for milk, bread, and butcher-meat are not subdivided. Very little butcher-meat requires to be kept, as it is brought in daily as required, but by a little rearrangement additional accommodation could, and should, be found for these articles. At the back entrance to the kitchen there is a most objectionable ashpit, which ought to be at once removed. There is plenty of room in the grounds to make suitable arrangements for this purpose, and the cost would be very small.

### Dispensary Department.

This building is centrally placed, and has good storage accommodation. At present some of the surgical dressings are kept in the matron's linen store, along with the general stock of linen. I would suggest that in future all surgical dressings, bandage cloth, surgical appliances, stationery, and drugs should be under the care of the apothecary. He should continue as at present to keep a record of all goods received into his department, but, in addition, each sister should be supplied with a book similar to the sample which I enclose, showing at a glance every article given out from day to day, with its cash value. This book should be submitted regularly to the matron and surgical staff for their information, and presented to the Committee once a month. By adopting this arrangement extravagance in the use of any article could be immediately detected, and checked, and a comparative record would be available at all times for reference.

### Need of an Identification Room.

The mortuary and adjoining rooms are well arranged, but there is no viewing room or chapel where relatives may identify and view the remains. A small chapel could be added to the end of the mortuary at very little cost. It would prove a valuable addition to the building, and would make a very suitable gift by any individual who wished to add to the efficiency of the institution.

### Secretarial Department and Collection of Funds.

The work in the secretarial department has been steadily increasing for some years. I would suggest that the secretary and his assistant be relieved of as much routine duty in the hospital as possible in order that they may be able to devote more time to the securing of the funds required to carry on the institution and the finding of money for the necessary extensions, which are so urgently required. The books are well kept, and go into great detail, but I would recommend the Committee seriously to consider the propriety of coming into line with other institutions of a similar nature, and adopt the uniform system of accounts, so that the cost of the staff and the patients may be separately detailed. They will find that this involves no additional labour. I would suggest also that in making contracts the details of each article be fully extended in every offer, so that when the articles come to be issued from the stores the cash value of each article may be entered opposite it in the requisition book. It seems to me that there is quite enough of work to keep a handy tradesman constantly employed, and I would recommend that every morning requisitions for structural repairs be sent to the secretary's office on forms supplied for the purpose duly initialled by the head of the department. The tradesman will take his instructions from the secretary, and every week the tradesman's diary should be written up by the assistant secretary and submitted to the Committee detailing the repairs and renewals requisitioned, also how and when they were carried out. Unless a regular system is adopted and someone is held responsible to the Committee, small matters are apt to be overlooked. I have considered how increased accommodation for patients might be obtained without interfering with the efficiency of the existing buildings, but these need not be detailed until this report has been considered and dealt with.

### Samaritan Society Suggested.

I would strongly recommend that a Samaritan Society should be established in connection with the Infirmary. At present certain articles are supplied to patients out of the Infirmary funds, such as surgical appliances, crutches, etc. These should not be chargeable against the cost per bed, but be procured through such an organisation as I suggest. I enclose you a copy of the objects of such a society, also a summary showing the amount of good work which can be overtaken in one year.

### Appeal to the Public.

If I can assist the Committee in carrying out any of the suggestions I have made with the object of increasing the efficiency and furthering the interests of the institution I am at their service, and I would urge on them the necessity of at once making an earnest appeal to the people of Bolton and neighbourhood to supply the necessary funds.—I am, ladies and gentlemen, your obedient servant,

Donald J. Mackintosh.

